# Idealized PPAR**γ**-Based Therapies: Lessons from Bench and Bedside

**DOI:** 10.1155/2012/978687

**Published:** 2012-06-14

**Authors:** Angélica Amorim Amato, Francisco de Assis Rocha Neves

**Affiliations:** Laboratório de Farmacologia Molecular, Departamento de Ciências Farmacêuticas, Faculdade de Ciências da Saúde, Universidade de Brasília, Campus Universitário Darcy Ribeiro, Brasília, CEP 70910-900, Brazil

## Abstract

The incidence of type 2 (T2D) diabetes and other chronic conditions associated with insulin resistance is increasing at an alarming rate, underscoring the need for effective and safe therapeutic strategies. Peroxisome-proliferator-activated receptor gamma (PPAR**γ**) has emerged as a critical regulator of glucose homeostasis, lipid homeostasis, and vascular inflammation. Currently marketed drugs targeting this receptor, the thiazolidinediones (TZDs), have proven benefits on insulin resistance and hyperglycemia associated with T2D. Unfortunately, they have been associated with long-term unfavorable effects on health, such as weight gain, plasma volume expansion, bone loss, cardiovascular toxicity, and possibly cancer, and these safety concerns have led to reduced interest for many PPAR**γ** ligands. However, over the last years, data from human genetic studies, animal models, and studies with ligands have increased our understanding of PPAR**γ**'s actions and provided important insights into how ligand development strategies could be optimized to increase effectiveness and safety of PPAR**γ**-based therapies.

## 1. Introduction

Peroxisome-proliferator-activated receptors (PPARs) are members of the nuclear receptor superfamily that heterodimerize with retinoid X receptors (RXRs) to modulate the transcription of target genes. They are activated by fatty acids [1] and are thus considered lipid sensors involved in the transcriptional regulation of energy metabolism [1]. Three isotypes of PPAR have been identified so far, namely, PPAR*α*, PPAR*β*/*δ*, and PPAR*γ*, each with a distinct pattern of tissue distribution and with unique physiological functions [2]. Briefly, PPAR*α* is found in the liver, kidney, heart, and muscle and is implicated in the uptake and oxidation of fatty acids and lipoprotein metabolism. PPAR*β*/*δ* is expressed in most cell types and plays an important role in lipid metabolism and cell differentiation and growth. PPAR*γ* actions are mediated by two isoforms, PPAR*γ*1, which has a wide tissue expression, and PPAR*γ*2, highly expressed in adipose tissue and considered the master regulator of adipocyte differentiation and function. It is noteworthy that PPARs are also expressed in macrophages, in which they are key modulators of the inflammatory response [3].

Consistent with their significance in metabolism physiology, this subfamily of nuclear receptors is an important target in metabolic disease. This is evidenced by the fact that PPAR*α* is the molecular target for the lipid-lowering fibrate drugs and PPAR*γ* is the target for the insulin-sensitizing TZDs. In fact, the identification of the lipid sensor PPAR*γ* as a key regulator of glucose metabolism came from the discovery that TZDs are potent agonists for this receptor [4]. TZDs increase insulin action in diverse animal models of insulin resistance and also in patients with T2D. However, the molecular basis of improved insulin sensitivity by activation of this “pro-obesogenic” receptor is incompletely understood [5], especially considering that obesity and T2D do not represent states of PPAR*γ* deficiency. Insights from tissue-specific animal knockout models of PPAR*γ* and also from ligand studies suggest there are at least two plausible mechanisms [6]. Activation of PPAR*γ* in adipose tissue improves its ability to store lipids, reducing lipotoxicity in muscle and liver. Also, PPAR*γ* agonists modulate the synthesis and release of a number of signaling molecules from the adipocytes and macrophages resident in the adipose tissue, with significant metabolic effects in other tissues [2]. There is also evidence that PPAR*γ* activation outside the adipose tissue is important for the insulin-sensitizing actions of TZDs [7–9].

Despite their metabolic benefits, TZDs may have clinically significant adverse effects, such as increased body weight [6, 10], fluid retention [11], increased risk of heart failure [11], bone loss [12], increased risk of myocardial infarction [13], and a potential link with bladder cancer [14, 15]. Because of the concerns on cardiovascular toxicity, rosiglitazone has been withdrawn in many countries worldwide, and due to concerns over its possible association with bladder cancer, pioglitazone has been suspended in some European countries.

These safety issues regarding TZDs have raised a number of questions. Firstly, what are the mechanisms underlying these unfavorable effects? Is PPAR*γ* still an attractive pharmacological target to treat metabolic disease? What are the tools to find safe and effective PPAR*γ* ligands? Over the last years, basic research and clinical studies have provided many insights into how PPAR*γ*-based therapies could be optimized.

## 2. What Are the Basis of TZDs' Adverse Events?

 Three TZDs have been approved for the treatment of insulin resistance associated with T2D over the last 15 years: troglitazone (which was discontinued in 1998), rosiglitazone, and pioglitazone (which have been discontinued in some countries and restricted in others). Although they are effective agents for the treatment of T2D, their use is associated with a number of adverse events. Some of them are considered common to the TZD class of drugs, whereas others are unique to individual TZDs. The latter are best characterized by idiosyncratic hepatotoxicity associated specifically with troglitazone [16], which was the reason for its discontinuance. Well-established class adverse effects include fluid retention, increased risk of congestive heart failure, weight gain and bone loss. The mechanisms underlying some of these unfavorable effects have been defined, but those of many others remain to be defined, as is the case of increased risk of myocardial infarction seen with rosiglitazone treatment [13] or the possible association between bladder cancer and pioglitazone [14, 15].

### 2.1. Fluid Retention

 TZD treatment is consistently associated with body fluid expansion, which is accompanied by hemodilution, peripheral edema, and the potential to increase the risk of congestive heart failure [11, 17]. The mechanisms underlying fluid retention are not completely defined, although PPAR*γ* action in modulating sodium transport in the collecting duct (CD) in both animal models [18, 19] and humans [20] seems to be involved. PPAR*γ* is mainly expressed in CD [21, 22] and CD-specific PPAR*γ* knockout in mice reduces fluid retention induced by TZDs [18, 19]. Moreover, activation of PPAR*γ* in CD cells results in increased expression of epithelium sodium channel (ENaC) [18, 19] and enhances apical localization of the *β*-subunit of the ENaC in cortical CD cells [23], which in turn increase sodium and fluid reabsorption. In addition, TZDs increase the activity of the ENaC and Na-K-ATPase system, independent of the increase in ENaC expression [24, 25]. There are also data to suggest that ENaC-independent mechanisms might be involved, since amiloride, an inhibitor of ENaC, fails to prevent TZD-induced fluid retention [24, 25]. Accordingly, aquaporin-2 has been also implicated in this phenomenon [26].

 Plasma volume expansion secondary to renal fluid reabsorption results in increase luminal pressure in the microvasculature, which in turn leads to a rise in pressure gradient across the microvessel wall and hence in fluid flux to the interstitial compartment [3]. This is considered as the main mechanism of formation of peripheral edema, although a direct action of TZDs in endothelium cells to increase vascular permeability, mediated by PPAR*γ*, has also been implicated [27–29].

 In addition to peripheral edema, renal fluid retention by TZDs is associated with the potential to increase cardiac load and precipitate or exacerbate congestive heart failure [30–32]. This has been the rationale to contraindicate TZD treatment in patients with class III or IV heart failure according to the criteria of the New York Heart Association [17]. Despite the propensity to precipitate congestive heart failure, there has been an intense debate over the possibility of direct cardiotoxicity of TZDs, especially of rosiglitazone, as will be discussed later.

### 2.2. Weight Gain

 Increases in body weight are seen with all TZDs in both animal studies including rodents and nonrodents [6] and clinical studies [10, 11]. This effect has been traditionally ascribed to increased adipogenesis and fluid retention resulting from PPAR*γ* activation by TZDs in adipose tissue and collecting duct cells, respectively. Moreover, it has been recently suggested that TZDs might influence energy balance by activating PPAR*γ* in the central nervous system (CNS) and inducing increased food intake [33, 34].

 Increased body fat mass has been classically associated with insulin resistance and cardiovascular disease, and hence weight gain is considered unfavorable in the treatment of T2D patients, in whom overweight or obesity is already frequent. However, increased adipogenesis with TZD treatment is associated with fat redistribution characterized by an increase in subcutaneous adipose tissue and concomitant decrease in visceral adipose tissue [35, 36]. Because of the unfavorable effect of visceral fat on insulin sensitivity, this redistribution of fat by TZDs is generally considered as beneficial in spite of increased body adiposity [37].

 Despite the correlation between increased insulin sensitivity and adipogenesis and fat redistribution by TZD treatment, the need for increased adipogenesis to the antidiabetic effect of these drugs has been questioned. A substantial part of the insulin-sensitizing effect of TZDs has been ascribed to their ability to induce adipocyte expression of adiponectin and reduce the expression other adipokines, which impair insulin action in peripheral tissues [2]. In addition, many PPAR*γ* ligands with partial agonist activity have been shown to dissociate adipogenesis and weight gain from the insulin sensitizing effects [38], as will be discussed later.

 Weight gain with TZD treatment has also been correlated with increased food intake for some years, at least in murine models [39]. Only recently, however, their effects on feeding have been dissociated from PPAR*γ* activation on the adipose tissue. Complimentary reports by two independent research groups have suggested that PPAR*γ* action in the CNS mediates its effects on food intake and energy balance [33, 34]. Ryan et al. showed that either acute or chronic activation of PPAR*γ* by TZD treatment or hypothalamic overexpression of PPAR*γ*, respectively, resulted in hyperphagia, positive energy balance, and weight gain. Conversely, inhibition of endogenous brain PPAR*γ* action led to the opposite effects [33]. Lu et al. demonstrated that neuron-specific PPAR*γ* knockout mice exhibited reduced food intake, increased energy expenditure during high-fat diet, resulting in reduced weight gain. Moreover, these animals were resistant to rosiglitazone-induced increase in feeding and weight gain [34].

### 2.3. Myocardial Infarction and Cardiovascular Mortality

 Increased risk of congestive heart failure with TZD treatment has been traditionally associated with the propensity of these drugs to induce plasma volume expansion and increased cardiac load. However, the role of PPAR*γ* in the heart has been controversial. Some animal studies have suggested that the direct action of PPAR*γ* on the heart could be beneficial, since TZDs improve cardiac performance [40, 41], decrease cardiac hypertrophy [42–44], and may also have beneficial effects on left ventricular remodeling and function after ischemic injury [45, 46]. Other studies, in contrast, have suggested that TZDs induce cardiac hypertrophy in rodent models of diabetes [47, 48], although increased cardiac mass could not be attributed directly to PPAR*γ* actions on the heart. Indeed, there are data to suggest that cardiac hypertrophy seen with TZDs may involve PPAR*γ*-dependent and independent pathways, since cardiomyocyte-specific PPAR*γ*-knockout mice were shown to develop cardiac hypertrophy and treatment of both wild-type and knockout mice with rosiglitazone also induced cardiac hypertrophy [49].

Clinical studies not primarily designed to address definite cardiovascular outcomes have also suggested no adverse effects of TZDs on cardiac performance or even a trend toward beneficial effects [40, 50]. Despite these potential favorable effects, in 2007 a meta-analysis indicated a significant increased risk for myocardial infarction and cardiovascular mortality in patients treated with rosiglitazone [13] and initiated concerns about the drug's cardiovascular safety. Since then, there has been no randomized controlled cardiovascular outcome trial sufficiently powered to confirm or refute these data [51–53]. Other meta-analyses conducted subsequently have either confirmed the initial findings or been inconclusive [54, 55], but none has refuted that rosiglitazone is associated with increased myocardial infarction risk. Moreover, the meta-analysis published in 2007 was updated in 2010 using alternative analysis to include trials with no cardiovascular events and confirmed the previous data that rosiglitazone increases risk for myocardial infarction [56].

 The concerns regarding rosiglitazone's cardiovascular safety have raised the question of whether pioglitazone treatment is associated with a similar risk, since the mechanisms underlying increased risk for myocardial infarction with rosiglitazone have not been defined and it is therefore not known whether they are specific to this drug or represent a class effect. The Prospective Pioglitazone Clinical Trial in Macrovascular Events (PROACTIVE trial) was a large randomized controlled trial designed to address cardiovascular outcomes that showed a benefit only in prespecified endpoints of death, myocardial infarction, and stroke [30]. It did not show statistically significant benefits in primary outcome, a broad composite of cardiovascular events. Smaller studies have similarly found that pioglitazone is not associated with increased cardiovascular risk other than the potential of exacerbation of congestive heart failure [57–59], whereas others have even suggested cardiovascular benefit [60].

 Collectively, these data have raised two important questions. Firstly, what are the potential mechanisms underlying the cardiovascular adverse effects associated with rosiglitazone treatment? Further, what explains the differences between rosiglitazone and pioglitazone with respect to cardiovascular hazards? These questions remain unanswered, although conceivable mechanisms have been suggested. Clinical studies have shown that pioglitazone and rosiglitazone have different effects on lipid profiles. Rosiglitazone treatment increases low-density lipoprotein cholesterol levels and triglyceride levels [61], whereas pioglitazone reduces triglyceride levels and induces greater increases in high-density lipoprotein cholesterol levels [61]. In addition, the pattern of modulation of gene expression seems to be different when comparing both TZDs [62–64]. In a murine model of diabetes, rosiglitazone upregulated the expression of a matrix metalloproteinase gene in the heart, which encodes an enzyme implicated in plaque rupture [64].

### 2.4. Bone Loss and Increased Fracture Risk

 Several clinical studies have linked both rosiglitazone and pioglitazone treatment to small but significant decreases in bone mineral density and increased fracture risk [12, 65–71], most frequently in women. Preclinical *in vivo* studies have greatly contributed to elucidate the mechanisms underlying this unfavorable effect. Treatment of mice with rosiglitazone suppresses osteoblast differentiation and increases marrow adipocytes [72], possibly by activating PPAR*γ* in bone marrow stromal cells and diverting them from the osteoblast lineage into the adipocyte lineage [73]. Marrow insulin growth factor system may also be involved, since it is a key modulator of osteoblast differentiation and proliferation, and activation of PPAR*γ* by rosiglitazone downregulates some components of this system [74]. Moreover, PPAR*γ* activation in hematopoietic precursors of the monocytic-macrophage lineage increases osteoclastogenesis and bone resorption [75].

### 2.5. Carcinogenesis

Concerns regarding the effect of TZDs on carcinogenesis are not recent; in 2005, pioglitazone and five of six dual PPAR*α*/*γ* agonists were listed as having carcinogenic activity in rat bladder, and this has been [76] the rationale for FDA's official requirement, since 2006, that 2-year rodent carcinogenicity studies with PPAR ligands are conducted before clinical trials [77]. These concerns have been intensified recently, after the publication of observational clinical studies linking pioglitazone to bladder cancer risk [14, 15]. In contrast, there have been no preclinical and clinical data linking PPAR*α* agonist to this type or cancer [78], neither there have been clinical data linking rosiglitazone to this type of cancer, although in a recent study rosiglitazone enhanced bladder tumors in rats pretreated with a bladder carcinogen [79].

Data from animal studies assessing the effects of PPAR ligands on tumorigenesis have been controversial. Some rodent studies have suggested that PPAR ligands may potentiate the development of diverse types of tumors, such as transitional cell carcinomas of the urothelium, hemangiosarcomas, liposarcomas, and sarcomatous tumors at various sites, whereas other animal studies have indicated a protective effect. These differences have been attributed to a number of factors, including ligand specificity (selective activation of PPAR*γ* versus activation of other PPAR isotypes), the animal model (rodent versus non-rodent), and cancer type [3]. This issue is further complicated by data from *in vitro* studies suggesting the antiproliferation properties of PPAR*γ* ligands [80]. Hence, the mechanisms underlying tumor formation are not established, and although the tumor types mentioned have been shown to express PPAR*γ* it still discussed whether these effects are receptor dependent or -independent.

In particular, urothelium carcinomas have been associated with pioglitazone and some dual PPAR*α*/*δ* agonists in different strains of rats (Sprague-Dawley, Fisher, Wistar). In these models, cellular hypertrophy has been an early finding in the bladder urothelium [81] although these effects have not been established as PPAR*γ*-dependent. In addition, there are data to suggest that these compounds may result in the production of cytotoxic urinary solids that could induce regenerative proliferation in the urothelium in rats [82]. However, this effect is not seen in mice and is not likely to occur in primates [82]. The significance of these findings to humans is not clear, but recent observations have linked pioglitazone to bladder cancer. An interim analysis of an ongoing 10-year observational study with diabetic patients has not indicated a significant risk of bladder cancer with pioglitazone treatment for a median duration of 2 years. However, this risk was significantly increased in patients with longest duration of drug exposure or highest cumulative drug dose [14]. Further, data from the Adverse Event Reporting System of the FDA and the French Agency for the Safety of Health Products indicated a significantly increased risk of bladder cancer with pioglitazone treatment [15]. Pioglitazone was then withdrawn in France and Germany, and regulatory agencies in other countries have recommended that the drug should not be used in patients with active bladder cancer [83]. Notwithstanding, in a cohort study of 252,467 patients with a followup of less than 6 years, pioglitazone was not associated with increased risk of cancer at various sites, including prostate, female breast, lung/bronchitis, endometrium, colon, pancreas, kidney/renal pelvis, rectum, and also of non-Hodgkin lymphoma and melanoma [84]. 

## 3. A Historical Perspective on the Concept of Safety and Efficacy of PPAR**γ** Ligands

The identification of PPARs as key regulators of diverse aspects of energy homeostasis has made them attractive pharmacological targets to treat metabolic diseases such as lipid disorders (drugs targeting PPAR*α* or -*δ*), T2D (drugs targeting PPAR*γ*), and obesity (drugs targeting PPAR*δ*).

Initial strategies of ligand design aimed to develop potent full agonists or ligands acting on different PPAR isotypes to broaden their therapeutic effects. With respect to drugs targeting PPAR*γ*, the clinical problems observed with the full agonists TZDs, as well as data from human genetic studies, animal knockout models, and preclinical and *in vitro* studies with ligands with different pharmacologic properties, have provided important insights into optimization of drug design strategies.

### 3.1. PPAR*γ* Ligand Specificity

 The possibility to target multiple risk factors associated with the metabolic syndrome by designing drugs with agonistic properties for more than one isotype of PPAR seemed very promising in the light of the diverse physiologic roles of this subfamily of nuclear receptors. Based on this rationale, some dual and pan-PPAR agonists were developed and some dual PPAR*α*/*γ* agonists were evaluated in clinical trials, including muraglitazar, tesaglitazar, ragaglitazar, MK-767, and imiglitazar [3]. Failure with these ligands is probably best exemplified by the first PPAR*α*/*γ* agonist, muraglitazar, which showed beneficial effects on glucose control and lipid levels of diabetic patients but was associated with a significantly increased risk of major cardiovascular events in a review of data from phase 2 and 3 clinical trials [85]. Other dual PPAR*α*/*γ* agonists evaluated in clinical trials were also discontinued due to safety concerns [3]. It should be noted, however, that the reason for development discontinuation of these drugs was always compound specific, and therefore it is not clear if their adverse effects are a class effects or are unrelated to PPAR activation.

 It is also noteworthy that the TZDs pioglitazone and rosiglitazone, although classically considered selective PPAR*γ* ligands [4, 86], show weak agonist activity in both PPAR*α* [87] and PPAR*δ* [87, 88]. In fact, the favorable effects of pioglitazone on lipid profile accounted for its agonist properties on PPAR*α* [89, 90]. As discussed before, although there are no data to attribute developmental failures with dual PPAR*α*/*γ* agonists to PPAR-dependent mechanisms, the properties of pioglitazone and rosiglitazone to activate both isotypes should be carefully considered.

### 3.2. Full versus Partial PPAR Agonists and Selective PPAR*γ* Modulation

 PPAR*γ* agonists can be grouped into full agonists, classically represented by the TZDs, and partial agonists that, at saturating concentrations, result in lower levels of receptor activation than that of a full agonists. The interest for compounds with partial agonist activity comes from better understanding of PPAR*γ* function with data from animal and human genetic studies and also from studies with ligands. The minor Ala allele of the human PPAR*γ*2 polymorphism Pro12Ala [91] results in reduced binding affinity for responsive elements and reduced transcriptional activity [92, 93]. Clinically, this allele has been associated with improved insulin sensitivity and reduced risk of T2D [94–96] and seems to be associated with increased weight [97]. In addition, mice with germline heterozygous deletion of the gene encoding PPAR*γ* resulting in reduced PPAR*γ* activity exhibited increased insulin sensitivity as compared to wild-type mice [98] and were also resistant to high-fat diet-induced obesity and insulin resistance [99]. Collectively, these findings suggest that milder degrees of PPAR*γ* activation, rather than its full activation, might be a better strategy to improve insulin sensitivity while preventing unfavorable effects of PPAR*γ* action [100]. Based on this concept, partial PPAR*γ* agonists are viewed as a strategy to maintain the benefits of PPAR*γ* activation and at the same time reduce dose-dependent side effects observed with the full agonists, such as weight gain and plasma volume expansion. Indeed, in animal models and clinical studies many compounds with weak agonist activity minimize these unfavorable effects without loss of the insulin-sensitizing and antidiabetic activity [101]. Due to their ability to discriminate between the actions of PPAR*γ* in different tissues, these compounds are also referred to as selective PPAR*γ* modulators (SPPAR*γ*M) [101].

 The molecular basis of the effects of SPPAR*γ*M is incompletely understood, but their effects probably stem from their distinct binding mode in the receptor's ligand binding pocket and differential recruitment of transcriptional cofactors [102], which can explain the different patterns of gene expression compared to that of full agonists [38]. However, the pattern of action of these ligands raise an important question: if the insulin-sensitizing and antidiabetic activity of PPAR*γ* is closely correlated with their ability to activate PPAR*γ*-induced transcription [86], why would ligands with weak agonist activity retain the favorable effects on glucose homeostasis, comparably to full agonists? Poor understanding of the mechanisms involved in the effects of partial PPAR*γ* actions may have been one of the reasons for the reduced interest in these compounds in clinical trials in spite of their favorable effects in *in vitro* and preclinical studies.

 A recent study by Choi et al. [103] greatly contributed to clarify important aspects of PPAR*γ* action. This work showed that obesity-related inflammation activates cyclin-dependent kinase 5 (Cdk5) in the adipose tissue, which phosphorylates PPAR*γ* at the serine residue at position 273 and results in dysregulation of a subset of PPAR*γ* target genes, with reduced expression of genes with favorable metabolic effects, notably insulin sensitivity. They also showed that both full and weak agonists inhibit PPAR*γ* phosphorylation by Cdk5 comparably. Moreover, this inhibition appears to be dissociated from classical receptor activation and is well correlated to the anti-diabetic effects of PPAR*γ* ligands. These data suggest the rationale behind the action of these ligands and may not only renew interest for partial PPAR*γ* ligands that have been already characterized *in vitro* and preclinically, but also be viewed as the basis for developing new PPAR*γ* ligands. It is important to note that these data also raise important questions. Firstly, how does Cdk5-mediated phosphorylation of PPAR*γ* lead to dysregulation of a subset PPAR*γ* target genes? Further, how can the binding of a ligand to PPAR*γ* inhibit S273 phosphorylation yet dissociate this effect from general transcriptional activity?

 Based on the concept that the transcriptional effects of PPAR*γ* ligands can be separated from the effects which result in insulin sensitization, in a subsequent work, Choi et al. [104] described a novel high-affinity synthetic PPAR*γ* ligand (SR1664) completely devoid of classical transcriptional agonism but with full blocking activity of Cdk5-medidated phosphorylation. Treatment of wild-type mice with obesity and insulin resistance induced by high-fat and high-sugar diet with this ligand resulted in improvement of insulin sensitivity but in a nonstatistically significant reduction in glucose levels. As expected, in cell-based assays SR1664 antagonized transcriptional activity of PPAR*γ* induced by rosiglitazone. Collectively, these data might indicate that a slight degree of partial agonism should be desirable for the benefits of PPAR*γ*-based therapies.

## 4. Concluding Remarks

 In the light of current knowledge regarding PPAR*γ* action, optimized ligands would be those with mild agonistic activity, potent phosphorylation-inhibiting activity, and tissue-specific actions. With this profile, it might be possible to lower the risk of side effects while achieving maximal efficacy in treating insulin resistance. An important question is whether it would be cost-effective to search for new ligands with these features, since there are safe drugs currently available to treat T2D. The answer is probably yes, since metformin is the only marketed drug to treat insulin resistance, an important physiopathological component of the disease. Moreover, insulin resistance is associated with conditions other than T2D, such as obesity, cancer, and cardiovascular disease, and therefore new insulin-sensitizing agents could potentially have extensive clinical indications.
